# Real-Time Bidirectional Pyrophosphorolysis-Activated Polymerization for Quantitative Detection of Somatic Mutations

**DOI:** 10.1371/journal.pone.0096420

**Published:** 2014-04-25

**Authors:** Najie Song, Xueting Zhong, Qingge Li

**Affiliations:** Engineering Research Centre of Molecular Diagnostics, Ministry of Education, State Key Laboratory of Molecular Vaccinology and Molecular Diagnostics, School of Life Sciences, Xiamen University, Xiamen, Fujian, China; University of Navarra, Spain

## Abstract

Detection of somatic mutations for targeted therapy is increasingly used in clinical settings. However, due to the difficulties of detecting rare mutations in excess of wild-type DNA, current methods often lack high sensitivity, require multiple procedural steps, or fail to be quantitative. We developed real-time bidirectional pyrophosphorolysis-activated polymerization (real-time Bi-PAP) that allows quantitative detection of somatic mutations. We applied the method to quantify seven mutations at codons 12 and 13 in *KRAS*, and 2 mutations (L858R, and T790M) in *EGFR* in clinical samples. The real-time Bi-PAP could detect 0.01% mutation in the presence of 100 ng template DNA. Of the 34 samples from the colon cancer patients, real-time Bi-PAP detected 14 *KRAS* mutant samples whereas the traditional real-time allele-specific PCR missed two samples with mutation abundance <1% and DNA sequencing missed nine samples with mutation abundance <10%. The detection results of the two *EGFR* mutations in 45 non-small cell lung cancer samples further supported the applicability of the real-time Bi-PAP. The real-time Bi-PAP also proved to be more efficient than the real-time allele-specific PCR in the detection of templates prepared from formalin-fixed paraffin-embedded samples. Thus, real-time Bi-PAP can be used for rapid and accurate quantification of somatic mutations. This flexible approach could be widely used for somatic mutation detection in clinical settings.

## Introduction

Somatic mutations in most cancers represent molecular signatures that are valuable for prognosis predication and treatment management. For example, the *KRAS* mutations in codons 12 and 13 are predictive markers of nonresponse to anti-epidermal growth factor receptor (anti-EGFR) antibodies like cetuximab [1] and panitumumab [2]. Mutations in *EGFR* can confer sensitivity or resistance to EGFR tyrosine kinase inhibitors such as gefitinib [3] and erlotinib [4] in patients with advanced non-small-cell lung cancer (NSCLC). However, detection of somatic mutations poses a technical challenge owing to the presence of large excess of wild-type DNA in tumor samples. This difficulty has been addressed by a wide range of molecular techniques for mutation detections. Methods commonly used include restriction enzyme digestion of wild-type DNA [5], [6], [7], peptide nucleic acids (PNA) suppression of wild-type elongation [8], [9], [10], [11], allele-specific amplification [12], [13], [14], sequence-specific ligation [16], and COLD-PCR [17]. More recently, digital PCR based on the compartmentalization and amplification of single DNA molecules [18], [19], [20] and deep sequencing based on next generation sequencing technology [21] are also proposed for increased sensitivity or multiplexity. However, most of these methods are inconvenient for use in clinical laboratories due to the insufficient selectivity, high costs, long turnaround time or complex manipulations.

In addition, with the advent of personalized medicine, there is a compelling need for quantitative measurement of somatic mutation level that may uncover critical pathological information in cancer studies. For example, a portion of patients characterized as being wild-type for *KRAS* fail to respond to anti-EGFR antibody therapy. One potential explanation is that some patients classified as wild-type for *KRAS* may have low, but clinically significant, levels of *KRAS* mutations. Currently, there is no evidence that small *KRAS* mutant subpopulations are affecting clinical outcomes with respect to EGFR-directed therapies. However, it will be necessary to quantitatively measure the levels of *KRAS* mutation to determine what level of *KRAS* mutation does predict failure to respond to therapies directed against the EGFR [22], [23]. In another example, circulating tumor DNA in plasma or serum could serve as a ‘liquid biopsy’ for numerous diagnostic applications and would avoid the need for tumor tissue biopsies [24]. One distinct feature of liquid biopsy is that it enables quantification of the mutant DNA levels. By taking repeated blood samples, the mutant level in circulating DNA can be traced during the natural course of the disease or during cancer treatment, allowing a precise monitoring of the disease status. Unfortunately, the difficulties in the detection of somatic mutations render the quantification an even more challenging task.

In clinical settings, samples of tumor tissue gathered during biopsy or resection are usually in the form of formalin-fixed paraffin-embedded (FFPE) diagnostic blocks, however, DNA templates prepared from FFPE tissues are of inferior quality as compared to their frozen counterparts owing to degradation of DNA caused by formalin fixation [25], [26]. Similar DNA fragmentation was also found in the circulating tumor DNA [27]. DNA fragmentation further narrows the range of choice for the detection methods to those only work on short DNA templates.

Pyrophosphorolysis-activated polymerization (PAP) is a method for highly specific nucleic acid amplification [28], [29], where pyrophosphorolysis and polymerization are serially coupled by DNA polymerase using PAP primers, which are blocked at their 3′ termini with dideoxynucleotides. By using bidirectional PAP (Bi-PAP), which is performed with a pair of opposing PAP primers that overlap by one nucleotide at their 3′ termini, great improvement in specificity could be achieved [30], [31]. So far, PAP or Bi-PAP works mainly in a polyacrylamide gel-electrophoresis mode, which is time-consuming, non-quantitative, and amenable to carry-over contamination. On the other hand, real-time PCR detection is a well-established approach, which features closed-tube working format and are characteristic of being rapid, simple as well as quantitative, and has been widely accepted in clinical settings.

In this study, we attempted to introduce real-time PCR detection into Bi-PAP to develop a highly selective yet rapid and quantitative method for detection of somatic mutations. We sought to (*a*) demonstrate the feasibility of the real-time Bi-PAP where Bi-PAP is carried out in a way like real-time PCR, (*b*) develop the multiplex approach for quantitative detection of somatic mutations by including an internal control, and (*c*) compare the results with the mainstream techniques in the detection of *KRAS* and *EGFR* mutations in clinical samples. Owing to the common use of FFPE samples, we also investigated the performance of this approach in the detection of FFPE-derived DNA.

## Materials and Methods

### Template DNA

Wild-type human genomic DNA was purified from the cultured human kidney 293T cells (ATCC CRL-11268, Manassas, VA) using a QIAamp DNA Mini kit (Qiagen, Valencia, CA). Purified DNA was quantified at absorbance of 260 nm using ND-1000 UV-VIS spectrophotometer (NanoDrop Technologies, Wilmington, DE), diluted into 10 mM Tris–HCl (pH 8.5) containing 1 mM EDTA to 20 ng/µl, and stored at −80°C before use. *KRAS* mutant templates were either purified from cell line SW480 (G12V, GGT>GTT, ATCC CCL-228) using the QIAamp kit (Qiagen) or were from artificial plasmids containing G12S (GGT>AGT), G12R (GGT>CGT), G12C (GGT>TGT), G12D (GGT>GAT), G12A (GGT>GCT) at codon 12 and G13D (GGC>GAC) at codon 13 purified from *E. Coli* DH5α. *EGFR* mutant templates were purified from cell line H1975 (ATCC CRL-5908) that harbors heterozygous L858R and T790M mutations. All the plasmids were linearized by cleavage with BamH 1 in order to obtain equivalent performance as genomic DNA [32].

### PAP Primers

PAP primers were designed using Primer premier 5.0 (PREMIER Biosoft International, Palo Alto, CA). Bi-PAP primers for seven *KRAS* mutations, two *EGFR* mutations, and the corresponding primers for internal controls were designed (Table S1). Three tags attached to the PAP primers were artificially generated and confirmed to have no significant similarity with any known human genomic sequences in NCBI through BLAST analysis (http://blast.ncbi.nlm.nih.gov/Blast.cgi). Three molecular beacons were designed according to the guidelines (www.molecular-beacons.com) to hybridize with the reverse complement of the tags. All the PAP primer precursors (without 3′-terminal dideoxynuclotide) and probes were synthesized by Sangon (Shanghai, China).

PAP primers were prepared from their precursors by adding 3′-terminal dideoxynuclotides through terminal deoxynucleotidyl transferase (TaKaRa Inc., Dalian, China) and purified by polyacrylamide gel electrophoresis. The quality of PAP primers was checked by determining the molecular weight of the oligonucleotides using Reflex III MALDI-TOF MS (Burker Daltonik, Bremen, Germany) followed by regular PCR at pH 8.3, where pyrophosphorolysis was inhibited. The PCR products should be negative with the PAP primers but positive with the PAP primer precursors [28], [29].

### Singleplex Real-Time Bi-PAP Assay

Singleplex real-time Bi-PAP assay was used to optimize the reaction conditions and to assess the sensitivity, specificity, and selectivity. The Bi-PAP reaction was performed in a total volume of 25 µl including 5 µl of DNA template, 50 mM Tris-HCl (pH 7.8), 16 mM (NH_4_)_2_SO_4_, 5.5 mM MgCl_2_, 25 µM each of dNTP, 90 µM Na_4_PPi, 2% dimethylsulfoxide (DMSO), 3 U of KlenTaq-S (Scientech Corp, St. Louis, MO), and optimized concentrations of primers as follows: 0.2 µM forward primer, 0.04 µM (G12V, G12S, G12R, G12D) or 0.08 µM (G12C, G12A, G13D) reverse primer, and 0.2 µM probe 1 for each mutation. The cycling conditions consisted of an initial denaturation step at 96°C for 3 minutes followed by 60 cycles of 95°C for 20 s, 60°C for 30 s, 64°C for 20 s, 68°C for 20 s, 72°C for 20 s. Fluorescence was monitored at 60°C in each cycle.

Serial dilutions of plasmid DNA of mutants containing 5, 50, 500, and 5000 copies per reaction, respectively, were used to determine the sensitivity of the reaction. Serial dilutions of wild-type human genomic DNA containing 1, 10, 100, and 500 ng per reaction, respectively, were used to assess the specificity of the reaction. Real-time Bi-PAP assays were carried out in Stratagene Mx3005p (Agilent technologies, Santa Clara, CA). The baseline and the quantification cycle (Cq) were set using the machine software version 4.10.

### Duplex Two-color Real-Time Bi-PAP Assay for *KRAS* Mutations

The reaction mixture consisted of 0.2 µM mutation specific forward primer, 0.04 µM (G12V, G12S, G12R, G12D) or 0.08 µM (G12C, G12A, G13D) reverse primer, 0.2 µM probe 1, 0.008 µM exon-4F, 0.04 µM exon-4R, 0.04 µM probe 3, and 5 µL of template containing certain percentage of mutation in the presence of 100 ng wild-type genomic DNA. All other components in the mixture as well as the cycling conditions were identical with the singleplex assay. In this reaction, primer exon-4F and exon-4R were used to amplify a region of exon 4 of *KRAS*, which was conservative and used as an internal control. To differentiate the target and internal control, probe 1 for mutation detection was labeled with FAM and the probe 3 for internal control detection was labeled with ROX. Quantification of mutant percentage was achieved through a calibration curve obtained by plotting the Cq difference between the mutation and the internal control (ΔCq =  Cq – Cq^IC^) with respect to the logarithmic mutant percentage.

Frozen tissue samples stored at −80°C were collected from 34 colorectal cancer patients in Zhongshan Hospital of Xiamen (Xiamen, Fujian, China). DNA was extracted from frozen tissues using QIAamp DNA Mini kit (Qiagen). For comparison, these samples were also detected by a commercial diagnostic kit AmoyDx *KRAS* (Amoy Diagnostics Inc., Xiamen, China), which is a real-time ARMS PCR assay. For confirmation, these samples were further analyzed by DNA sequencing using a pair of primers encompassing codons 12 and 13 of *KRAS*: 5′-AGGTACTGGTGGAGTATTTGATA-3′ (forward) and 5′-TTTATCTGTATCAAAGAATGGTCCT-3′ (reverse).

### Triplex Three-color Real-time Bi-PAP Assay for *EGFR* Mutations

The reaction mixture consisted of 0.2 µM L858R-F, 0.08 µM L858R-R, 0.2 µM T790M-F, 0.08 µM T790M-R, 0.12 µM Exon2-F, 0.024 µM Exon2-R, 0.2 µM probe 1, 0.2 µM probe 2, and 0.12 µM probe 3. All other components in the mixture as well as the cycling conditions were identical with the singleplex assay.

Frozen tissue samples stored at −80°C were collected from 20 NSCLC patients in Fujian Provincial Cancer Hospital (Fuzhou, Fujian, China). FFPE tissue samples were collected from 25 NSCLC patients in Zhongshan Hospital of Xiamen. DNA from frozen tissues was extracted as described above. DNA from FFPE tissues was extracted using QIAamp DNA FFPE Tissue kit (Qiagen). For comparison, these samples were detected by two commercial diagnostic kits AmoyDx *EGFR* (Amoy Diagnostics Inc) and TheraScreen *EGFR* RGQ PCR (Qiagen), both of which are real-time ARMS PCR assays. For confirmation, all these samples were analyzed by DNA sequencing using the following primers: 5′-CTTCTTCCCATGATGATCTGTC-3′ (forward) and 5′-AACAATACAGCTAGTGGGAAGG-3′ (reverse) for L858R; 5′-CGTAAACGTCCCTGTGCTA-3′ (forward) and 5′-CAGACCGCATGTGAGGAT-3′ (reverse) for T790M.

### Ethics Statement

The study protocol for sample collection was approved by Research Ethics Committee of Xiamen University and an informed consent was signed for each patient.

## Results

### Working Principle of Real-Time Bi-PAP

**Figure 1:**
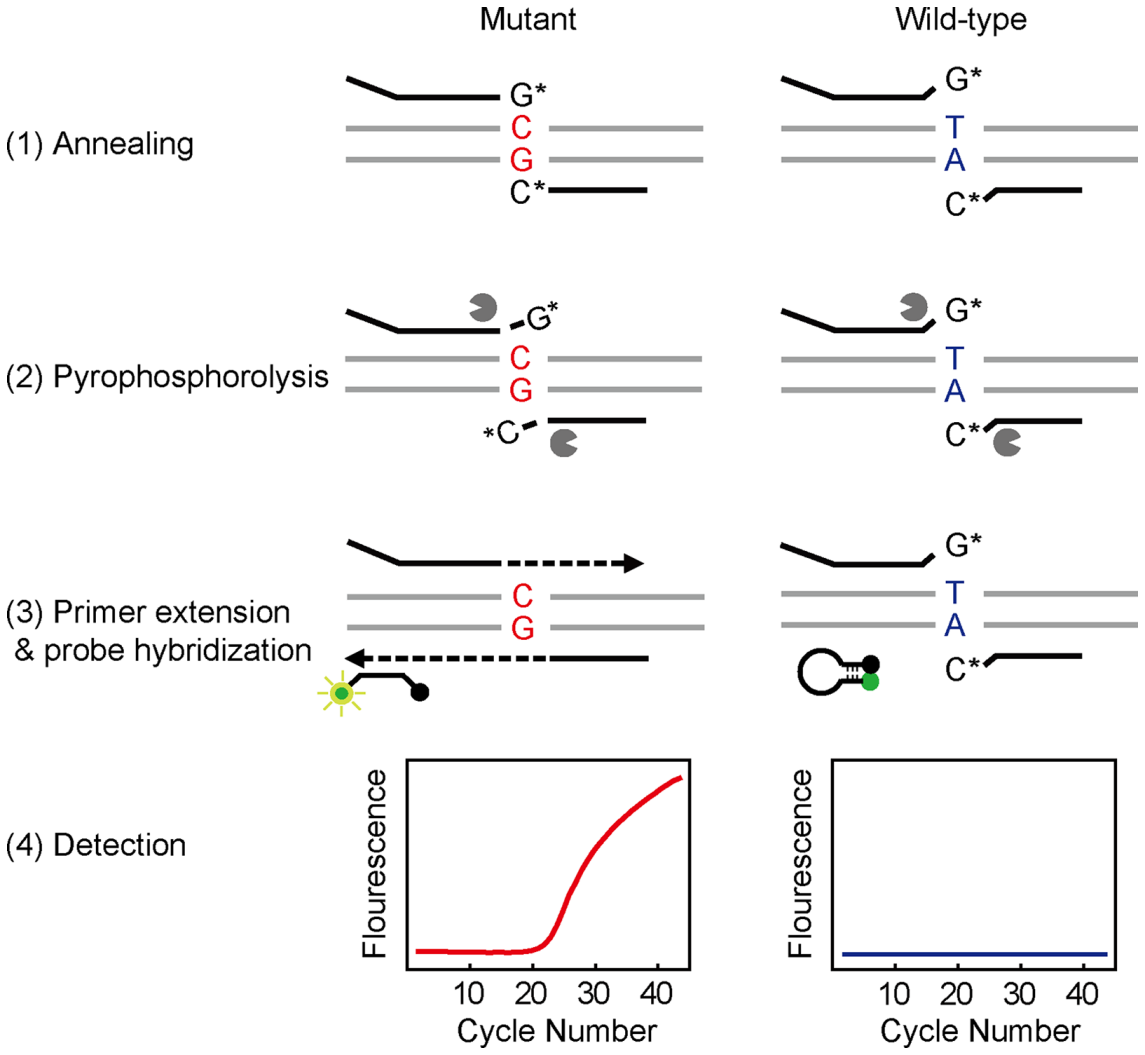
Working principle of real-time Bi-PAP. The working principle is schematically illustrated for the detection of a mutant template (shown as GC-containing, left panel) using a pair of Bi-PAP primers and a molecular beacon. The four steps: primer annealing, pyrophosphorolysis, primer extension/probe hybridization, and fluorescence detection, are shown from the top to bottom. As a result, fluorescence generated from the probe hybridization is detected in the form of amplification profile. In case of a wild-type template (shown as TA-containing, right panel), two Bi-PAP primers cannot be activated and extended owing to 3′-terminal mismatch, leading to no probe hybridization and thus no fluorescence signal.

Real-time Bi-PAP was designed by introducing a tag sequence to one of the Bi-PAP primers and a fluorogenic probe in the reaction can hybridize with the reversely complementary sequence of the tag (Figure 1). The working principle can be described as follows: 1) Annealing: Forward and reverse primers hybridize with the target DNA, resulting a fully matched hybridization for the mutant template and a 3′-terminal mismatch for the wild-type template. 2) Pyrophosphorolysis: The matched primers lose their 3′ terminal dideoxynucleotides catalyzed by the pyrophosphorolysis activity of KlenTaq-S and become unblocked whereas the 3′-termini mismatched primers stay blocked. 3) Primer extension and probe hybridization: The unblocked primers are elongated by the polymerase activity. After the extension is complete, the probe hybridizes with the reverse complement of the tag and becomes fluorescent. 4) Detection: Fluorescence is detected in each cycle and an amplification curve is generated. The wild-type template cannot be amplified because the two Bi-PAP primers remained blocked owing to 3′-terminal mismatch, leading to no extension for probe hybridization and thus no fluorescence signal.

### Working Conditions of Real-Time Bi-PAP

We first studied the working conditions of real-time Bi-PAP using *KRAS* G12R mutation as a model analyte. We followed the regular PAP working conditions but with different molar ratios of the tagged primer to the untagged primer (0.5∶5, 1∶5, 2∶5, 3∶5, 4∶5 and 5∶5). We observed that no amplification signals occurred when the amount of the two primers were equal. As the concentration of the tagged primer decreased, amplification signal became stronger. The strongest signal was obtained when the ratio was 1:5. Similar results were found in the detection of other mutations of *KRAS*. We therefore concluded that an asymmetric amplification with excess tagged primers is required for real-time Bi-PAP.

### Analytical Performance of Singleplex Real-Time Bi-PAP

**Figure 2:**
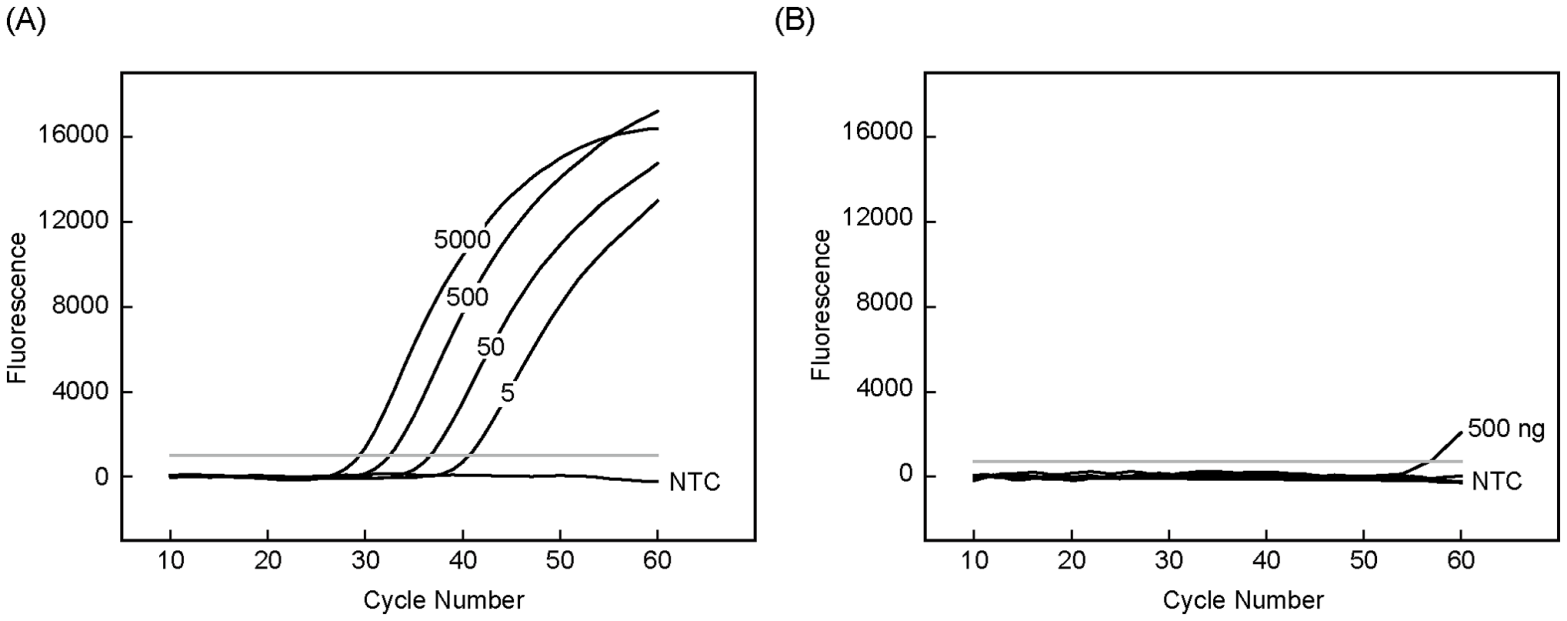
Study on the sensitivity and specificity of the singleplex real-time Bi-PAP. (A) Amplification curves of 5000, 500, 50, 5 copies per reaction (from the left to right) of G12R mutant plasmids. (B) Amplification curves of 500, 100, 10, 1 ng wild-type genomic DNA per reaction. Water was used as non-template control (NTC).

We evaluated the sensitivity, specificity, and selectivity of real-time Bi-PAP as defined for PAP [29]. Using *KRAS* G12R mutation as an example, we first determined the sensitivity with serial 10-fold dilutions of plasmid DNA in 10 replicates. The results showed that all dilutions could be detected repeatedly and the sensitivity was determined to be 5 copies per reaction (Figure 2). The specificity was tested with serial 10-fold dilutions of wild-type genomic DNA extracted from 293T cells in 10 replicates. The results showed that only 500 ng per reaction occasionally gave weak signals in the late cycles while all other dilutions consistently gave no signals. The specificity was thus determined to be 100 ng per reaction (∼30,000 copies per reaction). The selectivity was calculated by the ratio of sensitivity to specificity as 1.67×10^−4^. Similar results were obtained from other six *KRAS* mutations except for G12C, which had a specificity of 500 ng and selectivity of 3.3×10^−5^. The analytical performance of real-time Bi-PAP was comparable with a regular Bi-PAP [30].

### Two-color, Duplex Real-Time Bi-PAP Assay for *KRAS* Mutations

**Figure 3:**
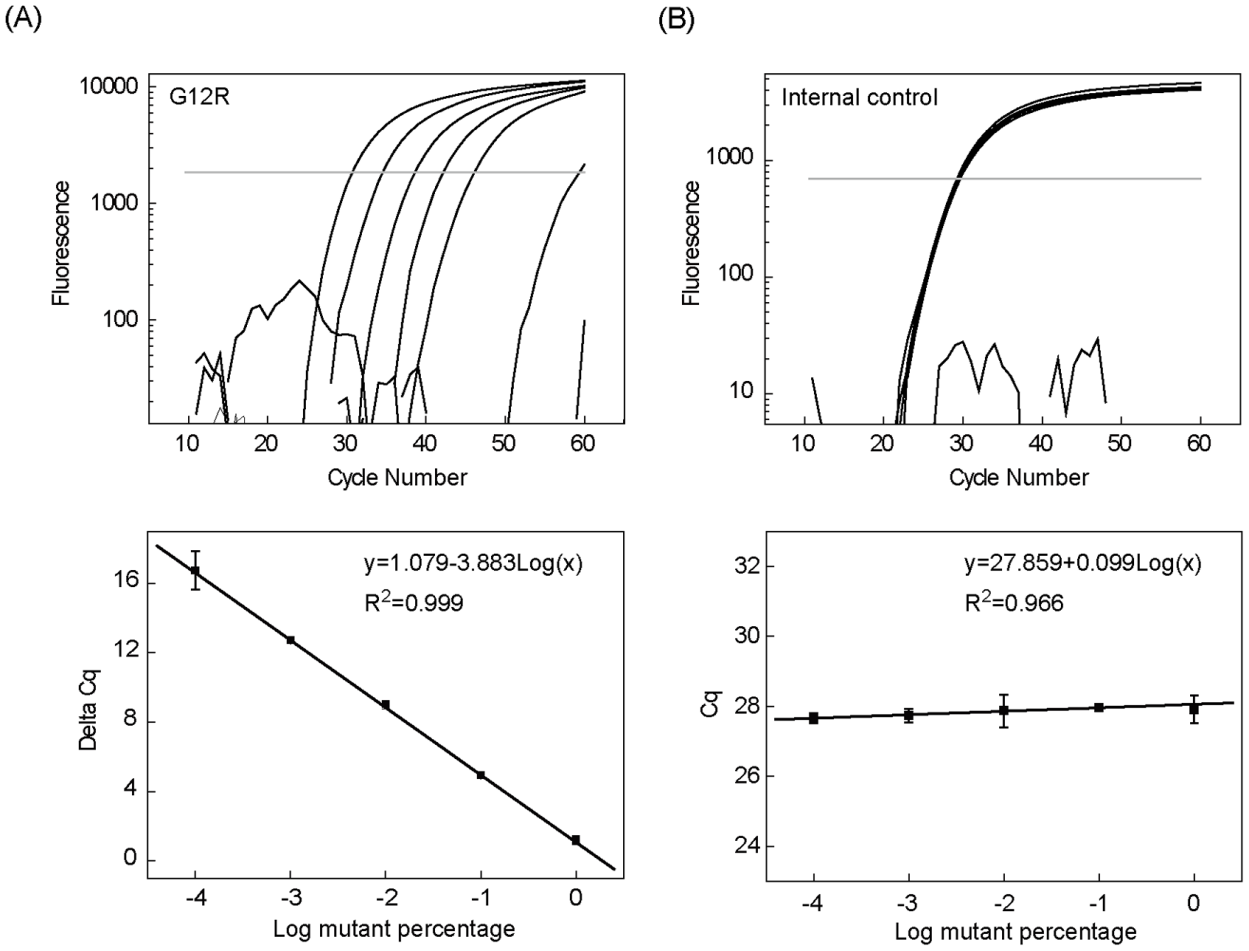
Quantitative performance of the two-color duplex real-time Bi-PAP. (A) Amplification curves of 100%, 10%, 1%, 0,1%, 0.01%, and 0% G12R mutant (from left to right) in the presence of 100 ng wild-type genomic DNA (upper panel). The linear relationship of the Cq difference between the mutation and the internal control (ΔCq =  Cq – Cq^IC^) with respect to the logarithmic mutation percentages (lower panel). (B) Amplification curves of the internal control with different mutant percentages (upper panel). The linear relationship between the Cq values of the internal control and the logarithmic mutant percentages (lower panel).

A two-color, duplex real-time Bi-PAP for *KRAS* mutations and an internal control was constructed for mutation quantification. We first studied the quantification range of this duplex Bi-PAP by serial 10-fold dilutions of mutant plasmids in the presence of 100 ng wild-type genomic DNA. By plotting the Cq difference between the mutation and the internal control (ΔCq =  Cq – Cq^IC^) with respect to the logarithmic mutation percentage from 100% to 0.01%, a linear relationship was achieved (Figure 3). Of note, the Cq^IC^ kept nearly constant regardless of the mutation percentages, demonstrating the negligible influence from the mutation target on the amplification of internal control, therefore ensuring the accuracy for mutation quantification.

Occasionally, we observed some non-specific, though weak, amplification signal from 100 ng wild-type DNA in the late cycles. During the experiments, we actually sequenced part of these false amplification products, and all of them had the sequence concordant with the PAP primers. These false results could exert influence on the limit of detection. To clarify their origin, we performed real-time Bi-PAP with both 100 ng wild-type genomic DNA and no-template control (NTC) in 10 replicates. The results showed that non-specific amplification signal only came from the wild-type genomic DNA samples but never from NTC. We thus concluded that the non-specific amplification was caused by “mis-priming” rather than primer dimer, which were not formed in real-time Bi-PAP. Similar results were observed from G12D, G12A, G12V, G12S, and G12R except from G12C and G13D where no false positive signals were detected.

**Figure 4:**
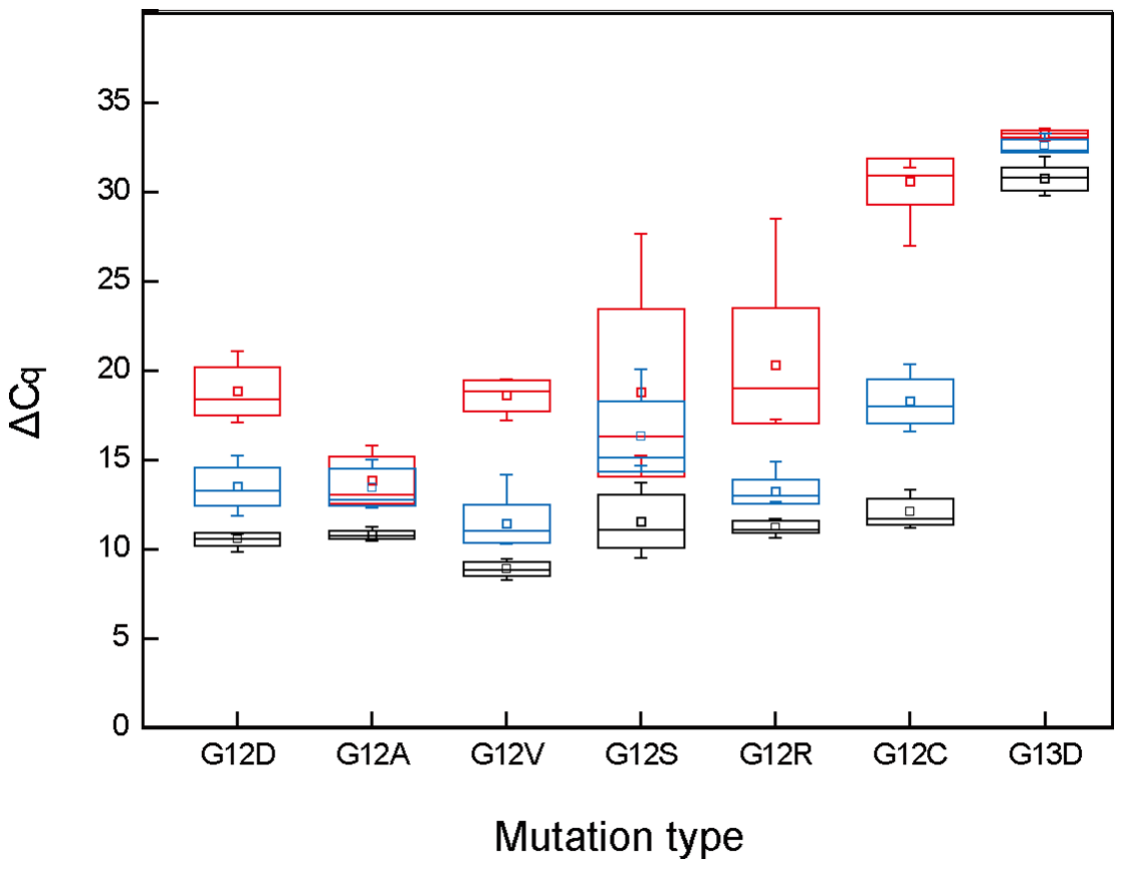
The limit of detection of the two-color duplex real-time Bi-PAP for each KRAS mutation in the presence of 100 ng wild-type genomic DNA. For each mutation, the ΔCq values for wild-type (red), 0.01% mutant (blue), and 0.1% (black) were detected in 10 replicates and calculated. The line within the box denotes the median, the square within the box denotes the mean, the horizontal borders of each box denote the SD, and the whiskers denote the minimum and maximum.

To determine the limit of detection, we analyzed two mixtures containing 0.01% and 0.1% mutant for each mutation type of *KRAS* in 10 replicate and calculated ΔCq. The limit of detection should have a ΔCq value not overlapped with 100 ng wild-type DNA by means of either “mean±SD” or “minimum to maximum”. According to this prerequisite, the limit of detection was 0.1% for G12A, G12S, and G13D; 0.01% for G12D, G12V, G12R, and G12C, respectively (Figure 4).

We analyzed 34 frozen tissue samples collected from colorectal cancer patients. For comparison, these samples were also analyzed in parallel by DNA sequencing and the commercial kit. Of the 34 samples, 14 samples were mutant by real-time Bi-PAP. Of these 14 mutant samples 12 were concordantly identified by the real-time ARMS PCR kit. The two missed mutant samples had the mutant percentage (0.07% and 0.26%) lower than the limit of detection of the real-time ARMS PCR kit (1%). DNA sequencing only identified five samples with mutant percentage higher than 10%, while the other nine mutant samples (mutant percentage <10%) were all undetected (Table S2). The above results suggested that real-time Bi-PAP is the most sensitive method for *KRAS* mutation detection, followed sequentially by the real-time ARMS PCR kit and the DNA sequencing analysis.

### Three-color, Triplex Real-Time Bi-PAP Assay for *EGFR* Mutations

**Figure 5:**
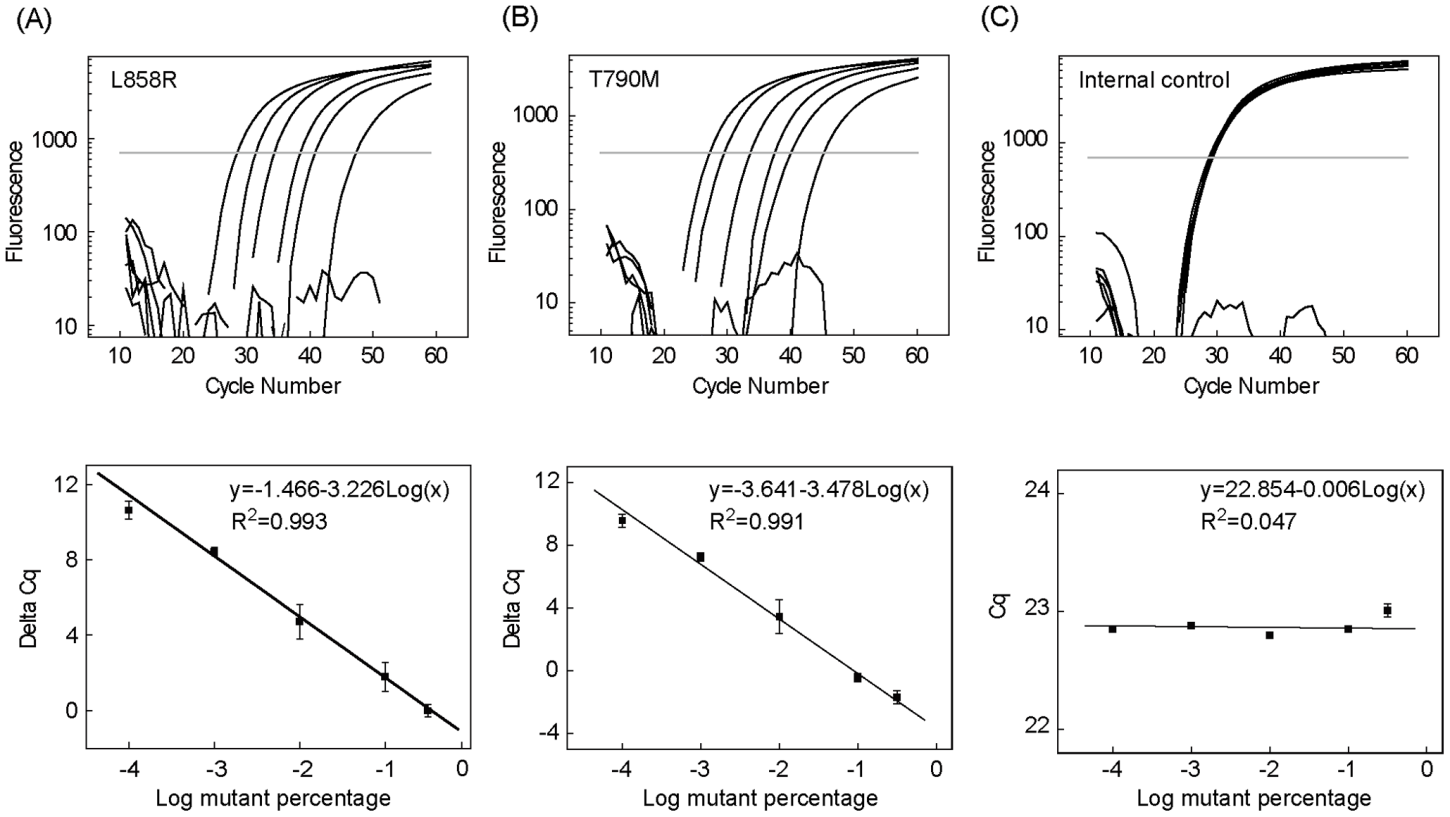
Quantitative performance of the three-color triplex real-time Bi-PAP. Amplification curves of 50%, 10%, 1%, 0.1%, 0.01%, and 0% mutant (from left to right) are shown for L858R (A) and T790M (B) in the upper panel. The linear relationship of the Cq difference between the mutation and the internal control (ΔCq =  Cq – Cq^IC^) with respect to the logarithmic mutation percentages are shown in the lower panel. Amplification curves of the internal control (C) with different mutant percentages are shown in the upper panel. The linear relationship between the Cq values of the internal control and the logarithmic mutant percentages is given in the lower panel.

A three-color, triplex real-time Bi-PAP assay was established for simultaneous detection of L858R, T790M, and an internal control. We first studied the quantification range of this triplex Bi-PAP by serial dilutions of mutant genomic DNA (from H1975 cell line) in the presence of wild-type genomic DNA (from 293T cell line) with the total amount of the template DNA kept at 100 ng. By plotting the Cq difference between the mutation and the internal control with respect to the logarithmic mutation percentage from 50% to 0.01%, a linear relationship was obtained (Figure 5). The limit of detection was determined using mixtures containing 0.1%, 0.01%, and 0% mutant genomic DNA with the overall template DNA kept at 100 ng. From 10 replicate detection results, the limit of detection was determined to be 0.01% for L858R and T790M.

Using the triplex three-color real-time Bi-PAP, we analyzed 20 frozen tissue samples and 25 FFPE tissue samples collected from NSCLC patients. For comparison, these samples were also analyzed in parallel by the two commercial diagnostic kits and DNA sequencing. Of the 45 samples, 10 samples were mutant by real-time Bi-PAP. These 10 mutant samples were also concordantly identified by the two real-time ARMS PCR kits. Among the 10 samples, DNA sequencing identified five samples that had relatively high mutant percentages (Table S3).

### Performance of Real-Time Bi-PAP in Analyzing FFPE-derived DNA

**Figure 6:**
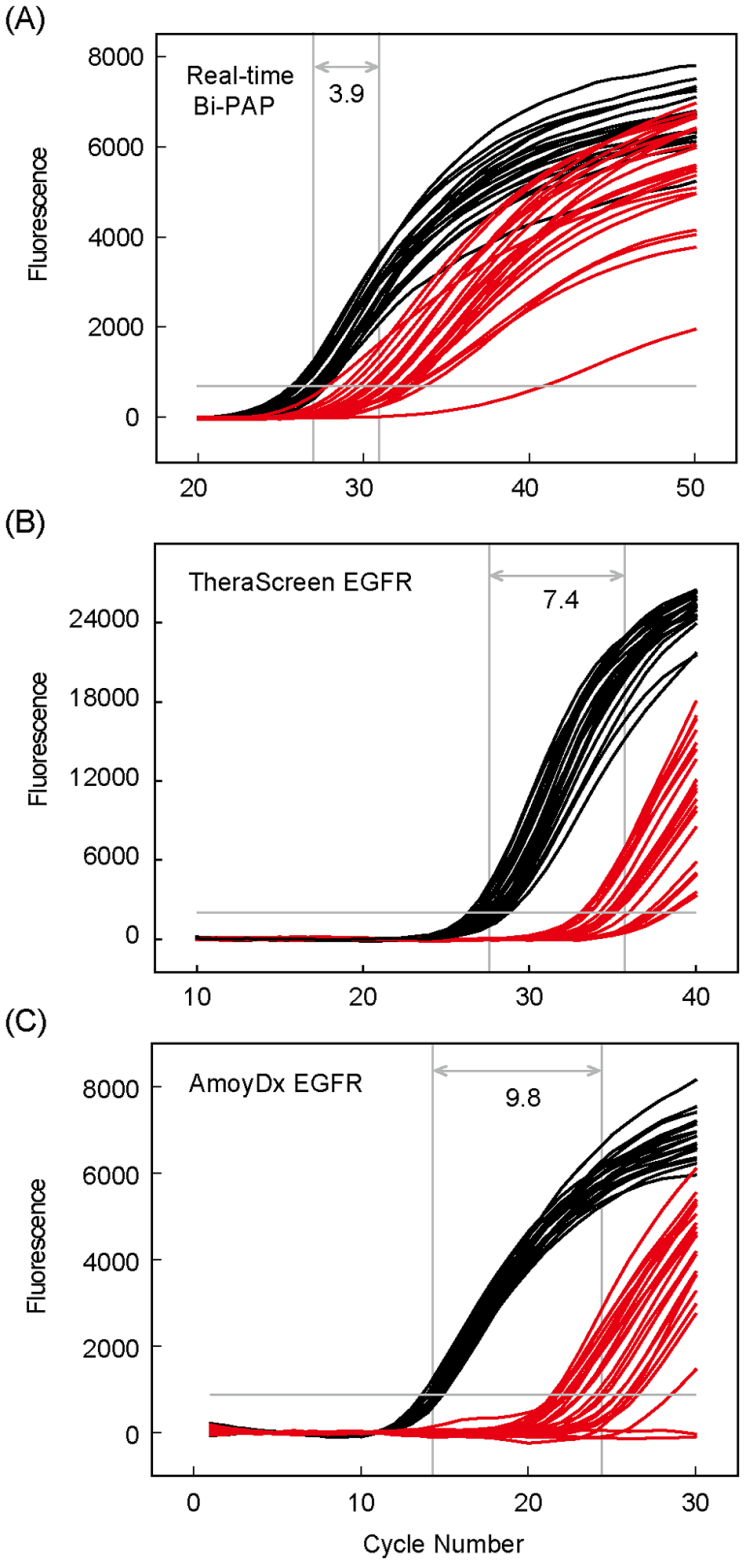
Performance comparison of real-time Bi-PAP and real-time ARMS PCR in the detection of template DNA derived from frozen tissue and FFPE tissue samples. (A) Real-time Bi-PAP. (B) TheraScreen *EGFR* RGQ PCR. (C) AmoyDx ARMS *EGFR*. Amplification curves shown are from 20 frozen tissue samples (black lines) and 25 FFPE tissue samples (red lines), respectively. The difference between the average amplification Cq values (indicated with a double-headed arrow) from the two types of samples are given for each assay in the detection of the internal control.

The template length of real-time Bi-PAP is one base shorter of the combined lengths of the two primers. Such a short template should render Bi-PAP more suitable for the fragmented DNA derived from FFPE tissue samples than those real-time ARMS PCR assays, which require much longer templates that need to encompass the binding regions for two primers and one probe. To test this assumption, we analyzed 25 FFPE samples and 20 frozen tissue samples using real-time Bi-PAP and the two real-time ARMS PCR kits, respectively. The amplification results from the internal control of each assay allowed a direct comparison between their performances. As shown in Figure 6, the frozen tissue-derived DNA template had earlier amplification signals than its counterpart FFPE tissue-derived DNA template in all cases. However, amplification cycles between the two sample types were very close with each other in real-time Bi-PAP whereas FFPE samples significantly lagged behind those from frozen tissue samples in the two real-time ARMS PCR kits. Calculation of the average Cq values from the two template types resulted in the smallest difference in real-time Bi-PAP. We therefore concluded that real-time Bi-PAP is more efficient than real-time ARMS PCR in the analysis of FFPE-derived DNA.

## Discussion

We developed the real-time Bi-PAP for quantification of somatic mutations. The uniqueness of this method is that pyrophosphorolysis, polymerization, probe hybridization, and fluorescence detection is accomplished in a closed tube and the mutation percentage can be quantified in a way like a standard real-time PCR. Therefore, combined advantages of Bi-PAP (extremely high specificity) and real-time PCR (quantification ability, rapidness and multicolor detection) are achieved. Moreover, the short input template required by real-time Bi-PAP increases its suitability with fragmented DNA derived from FFPE samples.

The high specificity of real-time Bi-PAP was demonstrated in the two examples of this study. In the detection of seven *KRAS* mutations, four mutations (G12D, G12V, G12R, and G12C) had a limit of detection of 0.01% and three other mutations (G12A, G12S, and G13D) had 0.1% in the presence of 100 ng wild-type template. For the two *EGFR* mutations (L858R and T790M), the limit of detection was 0.01% with both mutant types. This specificity level was close to the digital methods such as BEAMing [33] and digital PCR [20], and was at least 10-folds higher than the ARMS method. As demonstrated in the clinical studies, real-time Bi-PAP not only identified all those mutation-containing samples detected by the real-time ARMS kit and DNA sequencing, it also could identify those <1% mutant samples missed by the ARMS kit and DNA sequencing.

The quantification accuracy of real time Bi-PAP was ensured by the inclusion of an internal control in the reaction. The use of internal control for accurate gene quantification is well-established in real-time PCR [34]. In real-time Bi-PAP, the internal control represented the sum of wild-type and the mutant. The Cq^IC^ values could be used to indicate the reproducibility of the detection once the input template was constant. As shown in the *KRAS* and *EGFR* mutation detection, when the overall template DNA was 100 ng, the Cq values of the internal controls kept nearly constant regardless of the mutant percentages. This result suggested that the internal positive control could be independently amplified to embody the amount of the overall template. In this study, we kept the input template at 100 ng and used Cq =  Cq – Cq^IC^ as a measure to eliminate the well-to-well and sample-to-sample variations that may exist in quantification of different samples. These measures ensured the accuracy of the quantification.

Real-time Bi-PAP involved a single-step manipulation, and the entire procedure can be finished within 3 h. It is also cost effective when considering real-time PCR machine is now commonly used in most clinical molecular pathology laboratories. Finally, the results from the comparison study using frozen and FFPE samples showed that real-time Bi-PAP, which requires template no longer than the overall length of the two primers, was indeed more tolerable to potential fragmentation from FFPE-derived DNA than the real-time ARMS methods that rely on more intact template.

The above advantages of real-time Bi-PAP were also recognized in a recent study, where a dye-based real-time Bi-PAP assay was developed and validated for the detection of three tumor-specific mutations in circulating cell-free DNA of patients with uveal melanoma [35]. This assay allowed a quantitative detection of circulating tumor DNA in plasma from patients with metastatic uveal melanoma. The sensitivity and specificity in the detection of the three mutations obtained in this study were similar to those achieved in the present study. However, this dye-based real-time approach excluded the use of multiplex approach and therefore an internal control cannot be used for more precise quantification. In addition, the multiplexing detection cannot be further explored with this method.

The multiplexing ability of our approach may find flexible applications in clinical settings. Current real-time ARMS PCR often have more than 10 primers mixed together in a single reaction, enabling simultaneous detection of multiple mutations but at the cost of specificity restricted to 1–5% [36], [37]. According to ARMS, an error in primer recruitment would generate a template of the opposite allele, leading to exponential amplification of the error-containing template. In contrast, the specificity of real-time PAP is within 0.1–0.01%, which is at least 10-fold higher than the ARMS. This is because an error in PAP primer recruitment does not alter the intended template sequence and that the discrimination ability of the PAP primers kept unchanged during the entire amplification process [28]. As shown in this study, increasing the number of primer pairs in one reaction did not impair the analytical performance of either duplex or triplex Bi-PAP. This is due to the fact that Bi-PAP is completely free from primer dimer formation and has negligible mis-priming, and thus increasing primer number would not lead to more non-specific amplifications. Prospectively, the multiplexing ability of our approach might help build even higher order multiplexing detection.

There are two limitations for generally application of real-time Bi-PAP for rare mutation detection. One is the PAP primer preparation, which currently requires multiple reaction and purification steps. In our hand, starting from 10 nmol PAP primer precursors, the amount of PAP primers sufficient for 1,000 reactions can be prepared within 5 working days but with less than 4 h of hands-on time. The other limitation is its fairly moderate (10–100 fold) increase in detection sensitivity compared with ARMS and DNA sequencing. This makes real-time Bi-PAP not as sensitive as digital PCR in somatic mutation detection. However, since no special instrument is required for real-time Bi-PAP, the cost increase is marginal when compared with a standard real-time PCR while significant equipment and consumable saving can be achieved when compared with digital PCR.

In conclusion, we have demonstrated that real-time Bi-PAP can be used for the rapid and accurate quantification of somatic mutations. This flexible approach could be widely used for somatic mutations in clinical settings before digital PCR becomes commonly accessible and economically affordable.

## Supporting Information

Table S1 Primers and probes used in real-time Bi-PAP.
(DOCX)

Table S2 Summary of the detection results of *KRAS* mutations in frozen tissue samples by different methods.
(DOCX)

Table S3 Summary of the detection results of *EGFR* mutations in frozen tissue and FFPE tissues detected by different methods.
(DOCX)
