# Supplementary material for: Real-Time Bidirectional Pyrophosphorolysis-Activated Polymerization for Quantitative Detection of Somatic Mutations
Source: PLoS One. 2014 Apr 25;9(4):e96420. doi: 10.1371/journal.pone.0096420 (PMC4000192; doi:10.1371/journal.pone.0096420)
Supplement: Table S1 — Primers and probes used in real-time Bi-PAP. (DOCX) [file pone.0096420.s001.docx]

**Supplemental Data Table 1.** Primers and probes used in real-time Bi-PAP.

| Name | Sequence (5'-3') | Amplicon  Length (bp) |
| --- | --- | --- |
| G12S-F | ATGACTGAATATAAACTTGTGGTAGTTGGAGCTddA |  |
| G12S-R | Tag1-AGGCACTCTTGCCTACGCCACddT | 76 |
| G12R-F | ATGACTGAATATAAACTTGTGGTAGTTGGAGCTddC |  |
| G12R-R | Tag1-AGGCACTCTTGCCTACGCCACddG | 76 |
| G12C-F | ATGACTGAATATAAACTTGTGGTAGTTGGAGCTddT |  |
| G12C-R | Tag1-AGGCACTCTTGCCTACGCCACddA | 76 |
| G12D-F | TGACTGAATATAAACTTGTGGTAGTTGGAGCTGddA |  |
| G12D-R | Tag1-AAGGCACTCTTGCCTACGCCAddT | 76 |
| G12A-F | TGACTGAATATAAACTTGTGGTAGTTGGAGCTGddC |  |
| G12A-R | Tag1-AAGGCACTCTTGCCTACGCCAddG | 76 |
| G12V-F | TGACTGAATATAAACTTGTGGTAGTTGGAGCTGddT |  |
| G12V-R | Tag1-AAGGCACTCTTGCCTACGCCAddA | 76 |
| G13D-F | TGAATATAAACTTGTGGTAGTTGGAGCTGGTGddA |  |
| G13D-R | Tag1-CGTCAAGGCACTCTTGCCTACGddT | 76 |
| Exon4-F | Tag3-TTGCCTTCTAGAACAGTAGACACAAAACAddG |  |
| Exon4-R | GAATTCCATAACTTCTTGCTAAGTCCTGAGCddC | 84 |
| T790M-F | CTCACCTCCACCGTGCAACTCATCAddT |  |
| T790M-R | Tag2-GCCGAAGGGCATGAGCTGCddA | 65 |
| L858R-F | GCATGTCAAGATCACAGATTTTGGGCddG |  |
| L858R-R | Tag1-GCACCCAGCAGTTTGGCCddC | 66 |
| Exon2-F | ATCATTTTCTCAGCCTCCAGAGGATddG |  |
| Exon2-R | Tag3-AAGGACCACCTCACAGTTATTGAAddC | 73 |
| Tag1 | TCTCTCGCCACGCTCCCTCCG |  |
| Tag2 | CTCACTGTAGCACGTCGACC |  |
| Tag3 | ACAGCTCTGCACCCATCAACCTC |  |
| Probe1 | FAM-CGGAGGTCTCTCGCCACGCTCCCTCCG-Dabcyl |  |
| Probe2 | HEX-CCGGTCTCACTGTAGCACGTCGACCGG- Dabcyl |  |
| Probe3 | ROX-CCGAGGACAGCTCTGCACCCATCAACCTCGG-Dabcyl |  |
