# Supplementary material for: Real-Time Bidirectional Pyrophosphorolysis-Activated Polymerization for Quantitative Detection of Somatic Mutations
Source: PLoS One. 2014 Apr 25;9(4):e96420. doi: 10.1371/journal.pone.0096420 (PMC4000192; doi:10.1371/journal.pone.0096420)
Supplement: Table S2 — Summary of the detection results of KRAS mutations in frozen tissue samples by different methods. (DOCX) [file pone.0096420.s002.docx]

**Supplemental Table 2.** Summary of the detection results of *KRAS* mutations in frozen tissue samples by different methods

| Sample | Real-time Bi-PAP | | Sequencing | AmoyDx KRAS |
| --- | --- | --- | --- | --- |
|  | Type^a^ | Mutant (%)^b^ |  |  |
| 1 | WT | ND | WT | WT |
| 2 | WT | ND | WT | WT |
| 3 | G12R | 0.07 | WT | WT |
| 4 | WT | ND | WT | WT |
| 5 | G12D | 12.8 | G12D | G12D |
| 6 | G12V | 13.6 | G12V | G12V |
| 7 | WT | ND | WT | WT |
| 8 | WT | ND | WT | WT |
| 9 | WT | ND | WT | WT |
| 10 | WT | ND | WT | WT |
| 11 | G12V | 20.4 | G12V | G12V |
| 12 | G12C | 34.5 | G12C | G12C |
| 13 | WT | ND | WT | WT |
| 14 | G12D | 8.85 | WT | G12D |
| 15 | WT | ND | WT | WT |
| 16 | G13D | 12.3 | G13D | G13D |
| 17 | G13D | 2.7 | WT | G13D |
| 18 | WT | ND | WT | WT |
| 19 | G13D | 4.9 | WT | G13D |
| 20 | WT | ND | WT | WT |
| 21 | WT | ND | WT | WT |
| 22 | WT | ND | WT | WT |
| 23 | G12D | 2.85 | WT | G12D |
| 24 | WT | ND | WT | WT |
| 25 | G13D | 6.1 | WT | G13D |
| 26 | WT | ND | WT | WT |
| 27 | WT | ND | WT | WT |
| 28 | WT | ND | WT | WT |
| 29 | G13D | 0.6 | WT | G13D |
| 30 | WT | ND | WT | WT |
| 31 | G12R | 0.26 | WT | WT |
| 32 | WT | ND | WT | WT |
| 33 | WT | ND | WT | WT |
| 34 | G12V | 9.36 | WT | G12V |

^a^ WT, wild-type

^b^ND, not detectable
