# Supplementary material for: Real-Time Bidirectional Pyrophosphorolysis-Activated Polymerization for Quantitative Detection of Somatic Mutations
Source: PLoS One. 2014 Apr 25;9(4):e96420. doi: 10.1371/journal.pone.0096420 (PMC4000192; doi:10.1371/journal.pone.0096420)
Supplement: Table S3 — Summary of the detection results of EGFR mutations in frozen tissue and FFPE tissues detected by different methods. (DOCX) [file pone.0096420.s003.docx]

**Supplemental Table 3.** Summary of the detection results of *EGFR* mutations in frozen tissue and FFPE tissues detected by different methods.

| Sample | Source | Real-time Bi-PAP | | Sequencing | TheraScreen EGFR | AmoyDx EGFR |
| --- | --- | --- | --- | --- | --- | --- |
|  |  | Type^a^ Mutant (%)^b^ | |  |  |  |
| 1 | frozen | WT | ND | WT | WT | WT |
| 2 | frozen | WT | ND | WT | WT | WT |
| 3 | frozen | L858R | 10.3 | WT | L858R | L858R |
| 4 | frozen | WT | ND | WT | WT | WT |
| 5 | frozen | WT | ND | WT | WT | WT |
| 6 | frozen | WT | ND | WT | WT | WT |
| 7 | frozen | WT | ND | WT | WT | WT |
| 8 | frozen | WT | ND | WT | WT | WT |
| 9 | frozen | WT | ND | WT | WT | WT |
| 10 | frozen | L858R | 8.7 | L858R | L858R | L858R |
| 11 | frozen | L858R | 15.6 | L858R | L858R | L858R |
| 12 | frozen | L858R | 13.1 | L858R | L858R | L858R |
| 13 | frozen | L858R | 10.0 | L858R | L858R | L858R |
| 14 | frozen | L858R | 15.3 | WT | L858R | L858R |
| 15 | frozen | WT | ND | WT | WT | WT |
| 16 | frozen | L858R/T790M T790M | 8.4/1.3  1.3 | WT | L858R/T790M T790M | L858R/T790M  T790M |
| 17 | frozen | WT | ND | WT | WT | WT |
| 18 | frozen | WT | ND | WT | WT | WT |
| 19 | frozen | WT | ND | WT | WT | WT |
| 20 | frozen | L858R | 1.5 | WT | L858R | L858R |
| 21 | FFPE | WT | ND | WT | WT | WT |
| 22 | FFPE | WT | ND | WT | WT | WT |
| 23 | FFPE | WT | ND | WT | WT | WT |
| 24 | FFPE | L858R | 14.5 | WT | L858R | L858R |
| 25 | FFPE | WT | ND | WT | WT | WT |
| 26 | FFPE | WT | ND | WT | WT | WT |
| 27 | FFPE | WT | ND | WT | WT | WT |
| 28 | FFPE | WT | ND | WT | WT | WT |
| 29 | FFPE | WT | ND | WT | WT | WT |
| 30 | FFPE | WT | ND | WT | WT | WT |
| 31 | FFPE | WT | ND | WT | WT | WT |
| 32 | FFPE | WT | ND | WT | WT | WT |
| 33 | FFPE | WT | ND | WT | WT | WT |
| 34 | FFPE | L858R | 21.6 | L858R | L858R | L858R |
| 35 | FFPE | WT | ND | WT | WT | WT |
| 36 | FFPE | WT | ND | WT | WT | WT |
| 37 | FFPE | WT | ND | WT | WT | WT |
| 38 | FFPE | WT | ND | WT | WT | WT |
| 39 | FFPE | WT | ND | WT | WT | WT |
| 40 | FFPE | WT | ND | WT | WT | WT |
| 41 | FFPE | WT | ND | WT | WT | WT |
| 42 | FFPE | WT | ND | WT | WT | WT |
| 43 | FFPE | WT | ND | WT | WT | WT |
| 44 | FFPE | WT | ND | WT | WT | WT |
| 45 | FFPE | WT | ND | WT | WT | WT |

^a^ WT, wild-type

^b^ND, not detectable
